# Flavoring of sea salt with Mediterranean aromatic plants affects salty taste perception

**DOI:** 10.1002/jsfa.11953

**Published:** 2022-05-06

**Authors:** Antonella Rosa, Ilenia Pinna, Alessandra Piras, Silvia Porcedda, Carla Masala

**Affiliations:** ^1^ Department of Biomedical Sciences University of Cagliari, Cittadella Universitaria Monserrato Italy; ^2^ Department of Chemical and Geological Sciences University of Cagliari, Cittadella Universitaria Monserrato Italy

**Keywords:** salt reduction strategy, sea salt, taste, aromatic plants, flavored sea salt

## Abstract

**BACKGROUND:**

Salt (sodium chloride) is an essential component of daily food, crucial for many physiological processes. Due to health risks related to salt over consumption, considerable interest is devoted to strategies to reduce dietary salt intake. In this work we evaluated the sensory dimensions of sea salts flavored with Mediterranean aromatic plants with the aim to confirm the role of herbs/spices in the enhancement of salty perception and to validate the use of flavored salts as a strategy to reduce salt intake. To this goal we compared taste dimensions (pleasantness, intensity, and familiarity) of solutions obtained with salt and sea salts flavored with Mediterranean herbs, spices, and fruits. Sensorial differences were analyzed using a seven‐point hedonic Likert‐type scale on 58 non‐trained judges.

**RESULTS:**

Main flavor compounds, identified by gas chromatography‐flame ionization detection‐mass spectrometry (GC‐FID‐MS) analysis, were α‐pinene and 1,8‐cineole in myrtle salt (FS 1), verbenone, α‐pinene, 1,8‐cineole, and rosifoliol in herbs/plants salt (FS 2), and limonene in orange fruits/saffron salt (FS 3). At the dose of 0.04 g mL^−1^, saline solutions obtained with flavored salt (containing approximately 6–30% less sodium chloride) were perceived as more intense, less familiar, but equally pleasant than pure salt solution. In particular, sea salt flavored with orange fruits/saffron emerged as the most interesting in potentiating saltiness perception.

**CONCLUSION:**

Our study confirmed the important role of Mediterranean aromatic plants in the enhancement of saltiness perception and qualified the use of flavored sea salt during food preparation/cooking instead of normal salt as a potential strategy to reduce the daily salt intake. © 2022 The Authors. *Journal of The Science of Food and Agriculture* published by John Wiley & Sons Ltd on behalf of Society of Chemical Industry.

## INTRODUCTION

Salt (sodium chloride) is an essential component of daily food and is involved in regulating the water content (fluid balance) of the body, contributing to the body's homeostasis.^1^ Salt is a crystalline solid, white, normally obtained from seawater or rock deposits^2^ and used for human consumption as refined (table) salt, sea salt, flower salt, and processed salt.^2^, ^3^ Salt is a widely used food additive, both in industrial food processing and home cooking, for its preservative and antimicrobial properties^2^, ^4^ and its ability to increase liking of foods enhancing food flavor (flavoring agent).^4^, ^5^, ^6^, ^7^ The role of salt as food preservative in meat, seafood, and cheese is due to its ability to control bacterial growth, reducing water activity.^2^, ^4^, ^7^ Salt plays a role in enhancing the palatability of food flavor beyond imparting a desirable salty taste^4^, ^5^, ^6^, ^7^, ^8^ (salt flavor is one of the basic tastes), enhancing the perception of other aromatic compounds through cross‐modal interactions,^4^ and influencing the activity of some enzymes responsible for flavor development.^7^ In general, the presence of proteins, polysaccharides and lipids in foods reduces the volatility of aroma compounds, while the presence of salt increases volatility and allows flavor release and perception.^9^

An adequate intake of the sodium ion (Na^+^) is crucial for many physiological processes (generation of nerve impulses, renal function, regulation of acid–base balance and maintenance of blood volume and pressure).^1^, ^3^, ^4^, ^6^ However over consumption of salt through the diet is related to increased risk of hypertension, cardiovascular diseases, and renal insufficiency.^1^, ^3^, ^6^, ^10^ The World Health Organization recommended level of less than 5 g of salt intake per day in adults.^11^ Thus, considering the substantial role that salt taste plays in food acceptance/choice,^5^, ^6^ the food industry is looking for ways to reduce or replace salt in processed foods.^4^, ^5^, ^6^, ^12^, ^13^ Along with the search for salt substitutes,^4^, ^5^, ^7^, ^14^ actual research focuses on approaches that rely on modification or manipulation of salt taste.^5^, ^6^, ^7^, ^13^ Several studies were conducted to individuate flavor enhancers,^4^, ^6^, ^7^, ^12^, ^15^ compounds that do not taste salty itself but increase the taste intensity of a low amount of salt.^7^ In addition, other chemosensory systems (smell and chemesthesis) that contribute to overall flavor perception and play a crucial role in food acceptance, are actually taken into consideration in developing strategies to successfully reduce salt in the food supply.^5^, ^6^ The addition of high flavor ingredients (aromatic plants, mustards, and vinegars), during food cooking or manufacturing process, could assist in reducing the need to add salt.^4^, ^16^ There is actually a great interest in the dietary use of fresh herbs and spices for their ability to impart distinctive flavorings and positively affect health.^16^, ^17^, ^18^, ^19^ The addition of herbs and spices, that may sometimes be used instead of or in combination with added salt, has been suggested by many authors about strategies for reducing the amount of salt in the diet.^5^, ^16^, ^17^ Moreover, aromatic plants (spices/herbs) provide protein, fiber, volatile components (essential oils), vitamins, minerals and phytochemicals and greatly contribute for human health promotion due to their various properties (antioxidant activity, prevention of cancer, cardiovascular and neurodegenerative diseases).^16^, ^18^, ^19^

Salty, together with sweet, sour, bitter and umami, is a specific sensation of taste and saltiness is a specific sensation associated with a sodium chloride solution.^5^, ^6^, ^8^ Much research has been devoted to investigating and comparing the salty taste of different sea and land salts^20^, ^21^ in relation to their distinct chemical compositions, and the effect of the addition of spices on the taste properties of foods^19^, ^22^ and low‐salt food products,^17^, ^23^ confirming the role of aromatic plants in food saltiness enhancement. However, few research has been conducted on the quantification of the degree of saltiness enhancement by aromatic herbs via human taste sensory evaluation.^24^ Recently markets have emerged for gourmet types of salts such as Himalayan pink sea salt, premium Atlantic sea salt, and French grey sea salt,^25^ but also the consumption of salts flavored with aromatic herbs and spices^26^ has grown rapidly over the past 10 years for their strong impact on food flavor and palatability. Previous studies have been performed on the chemical composition and antioxidant properties of commercial mixtures of sea salt with spices/herbs.^26^, ^27^ However, to the best of our knowledge, no studies have been reported on the evaluation of the taste sensory properties of commercial sea salts flavored with dietary aromatic herbs and spices and on the role of these food ingredients in the enhancement of the salty taste perception in humans. Starting from all these considerations, in this study we assessed in humans the sensory dimensions (pleasantness, intensity, and familiarity) of sea salts flavored with herbs/plants normally included as spices or food/beverage in the Mediterranean region diet^26^ with the aim to confirm the role of aromatic herbs and spices in the enhancement of salty perception and to establish the use of flavored salts instead of normal salt as a strategy to reduce the daily salt intake. To this goal, we evaluated and compared the salty taste perception of saline solutions obtained with different types of commercial flavored sea salt with respect to pure salt (sodium chloride). Myrtle berries and leaves (product FS 1), a mixture of Mediterranean herbs and plants (helichrysum, rosemary, liquorice, fennel seeds, and myrtle leaves) (FS 2), oranges and saffron (FS 3) were used as salt flavoring ingredients. The taste of saline solutions was investigated determining the rate of the dimensions pleasantness, intensity, and familiarity^28^ using a hedonic scale method on 58 non‐trained judges. Quantitative analyses by gas chromatography‐flame ionization detection‐mass spectrometry (GC‐FID‐MS) of the main volatile compounds isolated from flavored salts were also performed and their potential contribution on the taste dimensions of flavored salt was evaluated.

## MATERIALS AND METHODS

### Chemicals

**Figure 1:**
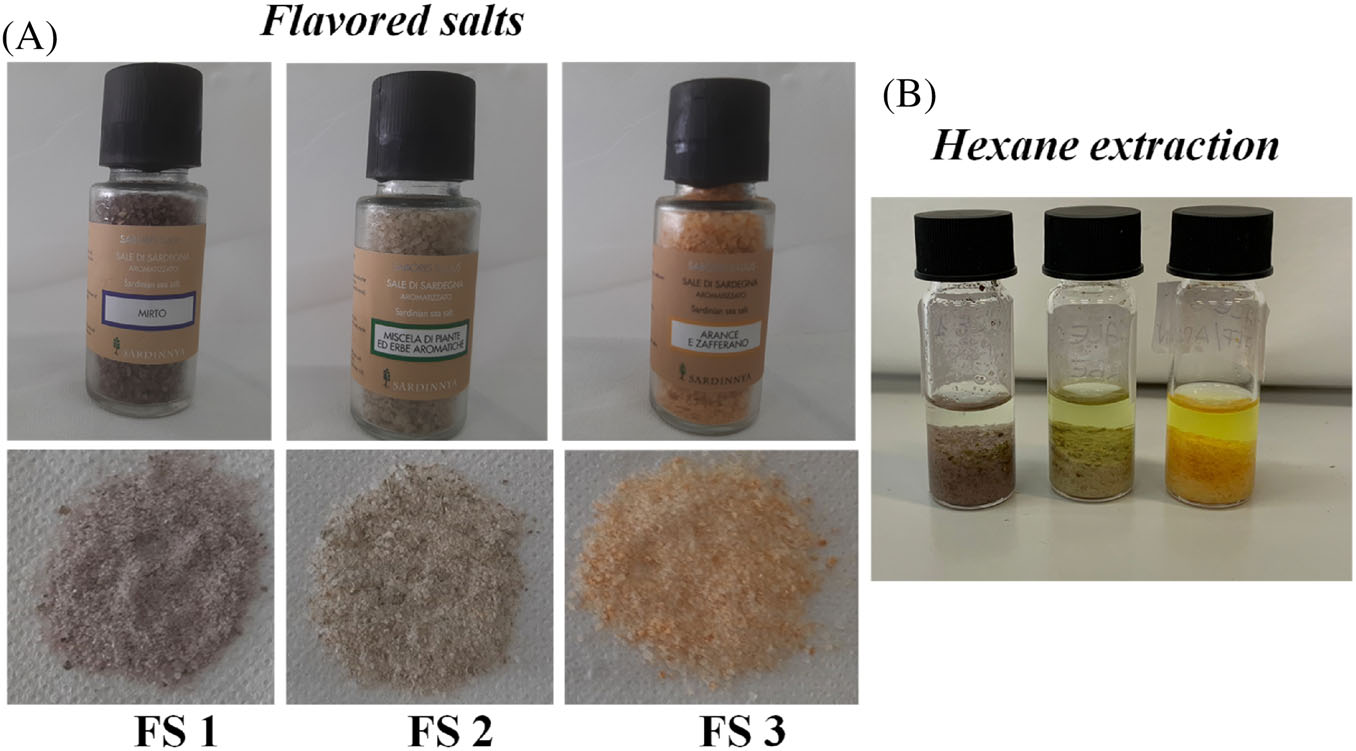
Digital images of commercial flavored sea salts of myrtle (FS 1), Mediterranean herbs and plants (FS 2), and orange and saffron (FS 3) (A). Cold maceration of flavored sea salts in *n*‐hexane (B).

Sodium chloride (purity ≥ 99.5%) for sensory assessment was purchased from Sigma‐Aldrich (St Louis, MO, USA). Flavored sea salts of myrtle (FS 1), Mediterranean herbs and plants (FS 2) and orange and saffron (FS 3) (Fig. 1) were kindly supplied by the company ‘Bresca Dorada s.r.l.’ located in Muravera, CA, Sardinia (Italy).

### Preparation of flavored sea salts

The production of flavored salts was carried out on semi‐industrial scale by the company ‘Bresca Dorada s.r.l.’. Commercial flavored salts were prepared using salt grains of high purity produced in salt pans of Cagliari (Italy), and vegetable matrices (herbs, plants, and fruits) provided by several local producers (Sardinia). For products FS 1 and FS 2,^26^ fresh plant parts were cleaned and then gently chopped/pounded with a cutter (Speedy Cutter DS; Nilma, Parma, Italy) to obtain small thin pieces, taking care to avoid oxidation and preserve the color. Separation of seeds from fruit pulp was performed by sieving. The blend parts of herbs, plants and fruits released water and these water mixtures (extracts) were used for salt aromatization. For product FS 3, oranges were carefully selected and washed, and peels were manually removed and grinded. The aqueous extract obtained from the saffron powder by infusion in hot water was mixed with grinded peels and the orange juice, and the mixture was used for salt aromatization. For all products, the mixing and the absorption of extracts to the sea salt were conducted in a mixer where extracts were used in an adequate liquid state to be easily absorbed by salt crystals. The drying of salt/extract mixtures was conducted with a refrigerant cycle dehumidifier at low temperature (maximum temperature of 35 °C) to prevent the fragrance loss. Finally, the essential oil (for product FS 1), obtained through the traditional hydro‐distillation process, was mixed with dried flavored salt. Flavored course sea salts were then packaged in glass jars with screw cap equipped with a grinder. The composition per kilogram of product of flavored sea salts FS 1–FS 3, as indicated on the commercial labels, is reported in Table 1.

**Table 1:** Summary of different type of commercial flavored sea salt as indicated on the labels per kilogram of product

Sample	Commercial name	Composition per kg of product
FS 1	Salt of myrtle	Sardinian sea salt, extract of myrtle (water, berries and leaves of *Myrtus communis*) (60 g kg^−1^), myrtle essential oil
FS 2	Salt of Mediterranean herbs and plants	Sardinian sea salt, extract of mixed herbs (helichrysum, rosemary, liquorice, fennel seeds and myrtle leaves) (150 g kg^−1^)
FS 3	Salt of oranges and saffron	Sardinian sea salt, extract of oranges and saffron (peels and juice of oranges fruits, *Crocus sativus*) (300 g kg^−1^)

### Preparation of *n*‐hexane extracts from flavored sea salts

Before sensory assessment, flavored salts were ground to obtain fine grains and weighed. Samples were extracted by maceration, using *n*‐hexane as solvent. This non‐polar organic solvent is well recognized as suitable for the extraction of a wide class of flavor compounds^29^ without salt extraction. Briefly, 1 g of flavored salt was treated with 1 mL of *n*‐hexane in a glass screw‐cap vial. After 24 h at 25 °C in the dark, the *n*‐hexane extract was filtered to remove salt residue. Aliquots (1 μL) of flavored salt *n*‐hexane extracts were directly injected (to avoid volatile compounds losses) into the GC–MS system for the determination of the qualitative‐quantitative composition of the main volatile components.

### GC‐FID and GC–MS analysis of *n*‐hexane extracts from flavored sea salts

Quantitative analyses were performed on an Agilent 7890A GC (Agilent, Santa Clara, CA, USA) equipped with a FID and a 30 m × 0.25 mm inner diameter (i.d.) with a 0.25 μm stationary film thickness HP‐5 capillary column. Quantification of constituents was made by integration of GC‐FID peak areas without using the response correction factors. The individual volatile compound concentration was expressed as percent peak relative to the total peak area from GC‐FID analysis of each sample. Qualitative analyses were carried out in a gas chromatograph (Agilent 6890N) equipped with a 30 m × 0.25 mm i.d. with a 0.25 μm stationary film thickness HP‐5 ms capillary column (Agilent J&W), coupled with a mass selective detector with an electron ionization (EI) device and a quadrupole analyzer (Agilent 5973).^30^ The compounds of the samples were identified by comparing mass spectra fragmentation patterns with those of a computer library, and linear retention indices (RIs) were based on a series of C_8_ to C_26_ *n*‐alkanes homologous to those reported in the literature.^31^

### Untrained sensory panels

Fifty‐eight participants were enrolled in this study, 26 men and 32 women. Panelists were not subjected to selection or training. Tasting sessions took place in a sensory room with individual sessions. Inclusion criteria were the absence of chronic/acute rhinosinusitis and systemic diseases related to smell disorders, and neurodegenerative diseases as previously reported.^28^, ^32^ None of the participants was taking medications for allergies or other medical illnesses during 5 days before test. These conditions were checked by the examiner before the beginning of procedure. Age (in years), weight (in kilograms) and height (in meters) were collected, and gustatory function were assessed in all participants. Participants received an explanatory statement and gave their written informed consent to participate in the study. This study was approved by the local Ethics Committee (Prot. PG/2018/10157) and was performed according to the Declaration of Helsinki.

### Assessment of gustatory function

Gustatory function was evaluated using the ‘Taste Strips’ test (Burghart Messtechnik, Wedel, Germany), based on filter paper strips impregnated with four concentrations of tastant for each basic taste qualities: sweet, bitter, sour, and salty.^33^, ^34^ The concentrations used for the taste strips were: sweet: 0.4, 0.2, 0.1, 0.05 g mL^−1^ of sucrose; bitter: 0.006, 0.0024, 0.0009, 0.0004 g mL^−1^ of quinine hydrochloride; sour: 0.3, 0.165, 0.09, 0.05 g mL^−1^ of citric acid; salty: 0.25, 0.1, 0.04, 0.016 g mL^−1^ of sodium chloride.^33^ Drinking water was used as solvent. Before the test, the mouths of participants were rinsed with water. The score ranged from 0 to 16 and a score < 9 was considered hypogeusia.^34^ Only normogeusic subjects were subjected to sensory assessment of flavored salts.

### Preparation of saline solutions for sensory assessment

Before sensory assessment, flavored salts were ground to obtain fine grains and weighed. Saline solutions were obtained dissolving flavored sea salts in drinking water (at the concentrations of 0.1 g mL^−1^ and 0.04 g mL^−1^). Control salt solutions were obtained dissolving an equal weight of salt (sodium chloride) in drinking water. These concentrations were chosen because they represented the two intermediate concentrations preliminarily used in the Taste Strips test^34^ for the assessment of gustatory function and were well perceived by the majority of subjects. Saline solutions were then aliquoted in 2 mL disposable plastic test tubes for sensory analysis.

### Procedures to assess taste pleasantness, intensity, and familiarity of sea salt solutions

All participants were asked to evaluate the taste dimensions of different saline solutions with a hedonic scale method.^28^, ^35^ Aliquots of 2 mL of each saline solution were used for sensory assessment. The taste pleasantness, intensity, and familiarity of saline solutions were assessed using a seven‐point Likert‐type scale ranging from 0 – not at all – to 6: such as 0 = very unpleasant and 6 = very pleasant; 0 = not intense at all and 6 = very intense; 0 = not familiar at all and 6 = very familiar.^28^, ^35^ All participants may drink only water 1 h before the experiment and did not wear any scented products on the day of testing. Before the experiment participants rinsed their mouths with water. Eight samples were presented in each session (1.5 h) with a mandatory 5 min rest in between samples. A rinse protocol between samples consisted of two rinsings with water. The order of taste ratings was randomized. During the session, panelists evaluated and discussed eight different saline solutions and generated subjective sensory attributes, such as the presence of particular flavor note or aftertaste.

### Statistical analyses

Data were expressed as a mean ± standard deviation (SD). Evaluation of statistical significance of differences was performed using Graph Pad INSTAT software (GraphPad software; San Diego, CA, USA) and with the software package SPSS software version 23 for Windows (IBM, Armonk, NY, USA). Normal distribution of data was preliminary assessed using the Shapiro–Wilk test. For each taste dimension (pleasantness, intensity, and familiarity), multiple comparison of means groups (salt, FS 1, FS 2, and FS 3) was assessed by one‐way analysis of variance (one‐way ANOVA) followed by the Bonferroni Multiple Comparisons Test to substantiate statistical differences between groups. Whereas comparison of means between two groups (salt *versus* flavored salt) for each taste dimension was assessed by Student's unpaired *t*‐test with Welch's correction, which does not require the assumption of equal variance between populations. Values with *P* < 0.05 were considered significant.

## RESULTS AND DISCUSSION

### Main components in flavored sea salts

**Figure 2:**
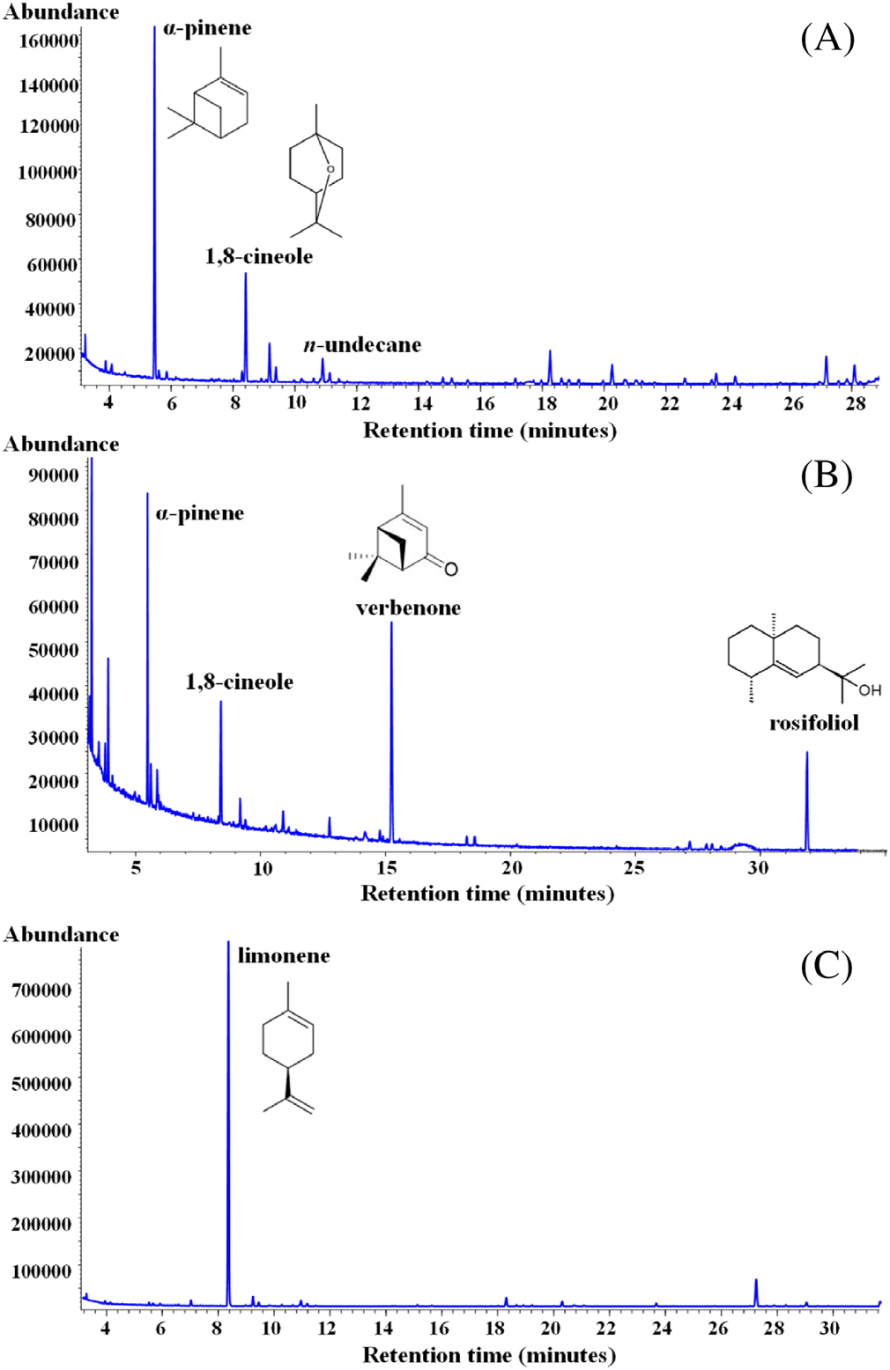
Chromatographic profiles by GC‐FID of *n*‐hexane extracts obtained from flavored sea salts of myrtle (FS 1) (A), Mediterranean herbs and plants (FS 2) (B), and orange and saffron (FS 3) (C). Chemical structures of the main volatile organic compounds are also reported.

Different types of sea salt flavored with extracts obtained from myrtle berries and leaves (FS 1), a mixture of Mediterranean herbs/plants (helichrysum, rosemary, liquorice, fennel seeds, and myrtle leaves) (FS 2), and oranges/saffron (FS 3) were subjected to a cold maceration with *n*‐hexane and extracts were analyzed by GC‐FID‐MS analysis. The use of *n*‐hexane as extracting solvent allowed to obtain non‐polar flavor compounds^29^ without salt extraction. *n*‐Hexane has been previously reported as proper solvent for the extraction of volatile compounds in honey without extraction of polar components like sugars and water.^36^ Flavored sea salts were characterized by a different amount of flavoring extract, that approximately accounted to 60, 150, and 300 g kg^−1^ in FS 1, FS 2, and FS 3, respectively, depending on the specific manufacturing process (Table 1). Figure 2 shows the chromatographic profile by GC‐FID analysis of flavored salt hexane extracts, with the indication of the main identified volatile compounds. Chemical analysis revealed that myrtle salt extract (FS 1) had a high content of the monoterpene hydrocarbon α‐pinene (33.3%) and the monoterpene cyclic ether 1,8‐cineole (13.1%), followed by lower amounts of different alkanes (*n*‐undecane 4%, hexadecane 3.2%, heptadecane 4.2%, octadecane 2.8%, and nonadecane 2.7%). α‐Pinene (∼10–60%) and 1,8‐cineole (∼12–34%) was previously reported as the most common volatile compounds found in myrtle leaves.^37^, ^38^ The *n*‐hexane extract obtained from herbs/plants salt (FS 2) was a very complex mixture, characterized by high amount of α‐pinene (15.3%), verbenone (15.8%), 1,8‐cineole (8.4%), and rosifoliol (5.4%), followed by lower amounts of different alkanes (mainly *n*‐undecane 5.1%, hexadecane 4.2%, and octadecane 3.7%). Rosemary essential oils were previously reported to be dominated by the aromatic compounds α‐pinene (13.5–37.7%), 1,8‐cineole (16.1–29.3%), and verbenone (0.8–16.9%),^39^ while rosifoliol was identified in the dried aerial parts of helichrysum.^40^ The main volatile component identified in FS 3 hexane extract was limonene, that accounted to 77.7% of the extract. Limonene has been indicated as the major compound (content ranges from 32% to 98% of the whole composition) of the essential oil extracted from orange (*Citrus sinensis*) peels (flavedo).^41^ Moreover, the yellow color of the FS 3 hexane extract evidenced the presence of saffron carotenoids.^42^ Saffron, the stigmas of the flower *Crocus sativus* Linnaeus, contains high amounts of fat‐soluble carotenoids (carotene, lycopene, and zeaxanthin) and water‐soluble carotenoids (apocarotenoid crocetin and crocins).^42^

Among polar components (Table 2), we have previously demonstrated the presence of several phenolic compounds in flavored sea salts.^26^ Gallic acid, myricitrin, ellagic acid, quercetin 3‐galactoside, quercetin 3‐glucoside, quercitrin, myricetin, and myricetin‐3‐galactoside were the main phenolics identified in myrtle sea salt (FS 1).^26^ The phenolic diterpenes carnosol and carnosic acid, the flavonoid diosmin, rosmarinic acid, and gallic acid emerged as the most abundant compounds in mixed herbs/plants salt (FS 2), whereas the flavonoids hesperidin neohesperidin, nobiletin, and tangeretin were the main phenols present in sea salt flavored with citrus fruits.^26^ Proton nuclear magnetic resonance (^1^H NMR) spectroscopy previously evidenced in FS 1 and FS 2 and citrus fruits salt (Table 2) the presence of carbohydrates, organic acids, and amino acids.^26^

**Table 2:** Literature data^26^ on chemical composition of different type of flavored sea salt

Flavored salt	Identified compounds
Salt of myrtle	Ellagic acid, gallic acid, myricetin, myricetin‐3‐galactoside, myricitrin, quercetin 3‐galactoside, quercetin 3‐glucoside, quercitrin, vitexin, β‐glucose, α‐glucose, fructose, asparagine, citric acid, quinic acid
Salt of Mediterranean herbs and plants	Carnosic acid, carnosol, diosmin, ellagic acid, eriocitrin, gallic acid, hesperidin, luteolin, luteolin 7‐glucoside, methyl carnosate, myricitrin, nobiletin, rosmarinic acid, β‐glucose, α‐glucose, fructose, alanine, citric acid, acetic acid, quinic acid
Salt of citrus fruits^a^	Diosmin, eriocitrin, hesperidin, isorhoifolin, luteolin 7‐glucoside, myricitrin, narirutin, neohesperidin, nobiletin, rhoifolin, tangeretin, naringin, β‐glucose, α‐glucose, fructose, alanine, asparagine, acetic acid

^a^ The product SF 3 differed for the presence of saffron extract.

**Table 3:** Mean values ± standard deviation of age, height, weight, body mass index (BMI), and gustatory function (sweet, salty, sour, and bitter) determined in all participants (*n* = 58), men (*n* = 26), and women (*n* = 32)

Parameters	All participants	Men	Women
Age (years)	31.48 ± 15.27	30.88 ± 15.91	31.71 ± 15.91
Height (m)	1.65 ± 0.08	1.73 ± 0.09	1.62 ± 0.06
Weight (kg)	63.84 ± 13.02	75.56 ± 12.60	59.38 ± 10.18
BMI	23.36 ± 4.06	25.44 ± 4.84	22.57 ± 3.46
Gustatory function	All participants	Men	Women
Sweet taste	3.71 ± 0.56	3.56 ± 0.63	3.78 ± 0.53
Salty taste	3.55 ± 0.63	3.63 ± 0.62	3.53 ± 0.64
Sour taste	2.45 ± 0.95	2.19 ± 0.91	2.55 ± 0.96
Bitter taste	3.05 ± 1.00	3.19 ± 1.05	3.00 ± 0.99
Total taste	12.77 ± 1.67	12.56 ± 1.55	12.85 ± 1.73

### Sensory properties of flavored sea salts *versus* salt

**Figure 3:**
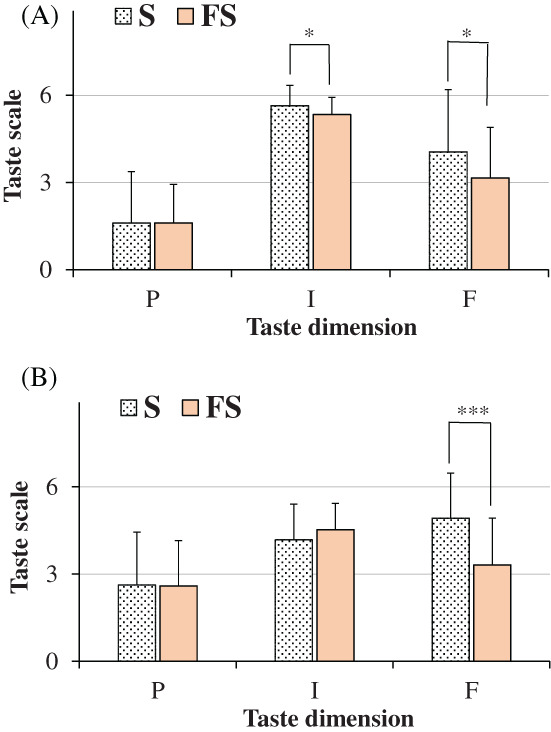
Ratings of pleasantness (P), intensity (I), and familiarity (F) dimensions determined for the taste of saline solutions obtained with salt (S, 100% sodium chloride) and flavored sea salt (FS) at the concentrations of 0.1 g mL^−1^ (A) and 0.04 g mL^−1^ (B). Data are presented as mean values and standard deviations (*n = 58*). For FS, each value of taste dimensions was obtained as mean of participant responses to three different commercial FS samples. ***P < 0.01, *P < 0.05 (Student's unpaired *t*‐test with Welch's correction).

**Figure 4:**
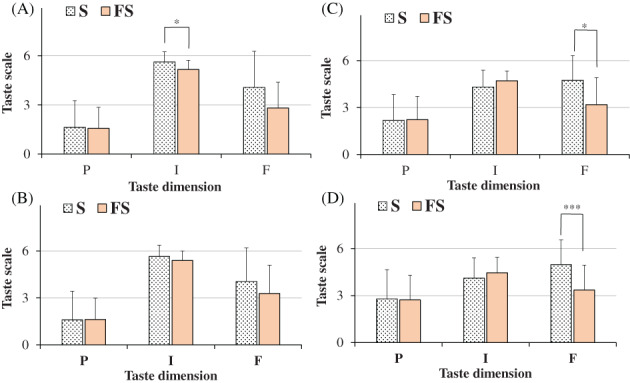
Ratings of pleasantness (P), intensity (I), and familiarity (F) dimensions determined for the taste of saline solutions obtained with salt (S, 100% sodium chloride) and flavored sea salt (FS) at the concentrations of 0.1 g mL^−1^ in men (*n* = 26) (A) and women (*n* = 32) (B) and 0.04 g mL^−1^ in men (*n* = 26) (C) and women (*n* = 32) (D). Data are presented as mean values and standard deviations. For FS, each value of taste dimensions was obtained as mean of participant responses to three different commercial FS samples. ***P < 0.01, *P < 0.05 (Student's unpaired *t*‐test with Welch's correction).

Mean values ± SD of age, height, weight, and gustatory function for all participants (untrained panelists), men and women were indicated in Table 3. An age range 18–79 years and a mean age ± SD of 31.48 ± 15.27 were determined for all subjects (*n = 58*). Men and women showed similar mean values of age, height, weight, and gustatory function. Figure 3 showed the ratings of pleasantness, intensity, and familiarity dimensions determined for the taste of saline solutions obtained with salt (S, 100% sodium chloride) and flavored sea salt (FS) at concentrations of 0.1 g mL^−1^ (high dose, Fig. 3(A)) and 0.04 g mL^−1^ (low dose, Fig. 3(B)), considering all participants together. The chemosensory response of each participant to flavored salt was reported as mean value ± SD of the scores for the three different flavored salts. At the high dose, mean values were 1.60 ± 1.77, 5.64 ± 0.69, and 4.05 ± 2.14 for salt taste pleasantness, intensity, and familiarity, respectively. At the low dose, higher scores were measured for salt taste pleasantness (2.62 ± 1.81) and familiarity dimensions (4.91 ± 1.56), with a lower intensity rate (4.17 ± 1.23), with respect to the high dose. Flavoring induced a modulation of salty perception. Saline solutions obtained with flavored sea salt showed, at both doses, similar rate of taste pleasantness than salt, but taste was significantly less familiar. Unless at high dose taste intensity of flavored sea salt was slight lower than salt, interestingly at low dose the taste intensity was higher in flavored sea salt than salt. Similar ratings of pleasantness, intensity, and familiarity dimensions were determined at both doses in men and women for the taste of saline solutions obtained with salt and flavored salt (Fig. 4).

**Figure 5:**
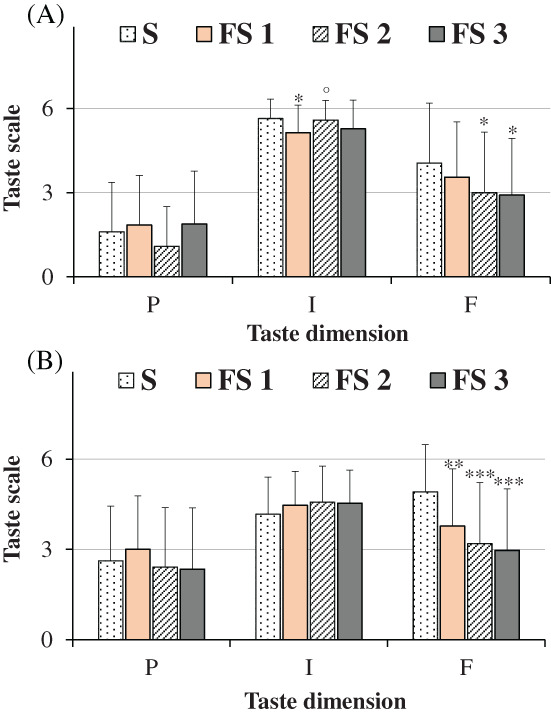
Ratings of pleasantness (P), intensity (I), and familiarity (F) dimensions determined for the taste of saline solutions obtained with salt (S, 100% sodium chloride) and flavored sea salts of myrtle (FS 1), Mediterranean herbs/plants (FS 2), and orange/saffron (FS 3) at the concentrations of 0.1 g mL^−1^ (A) and 0.04 g mL^−1^ (B). Data are presented as mean values and standard deviations (*n* = 58). ***P < 0.001, **P < 0.01, *P < 0.05 *versus* S, °*P* < 0.05 *versus* FS 1 (one‐way ANOVA and the Bonferroni *post hoc* test).

**Figure 6:**
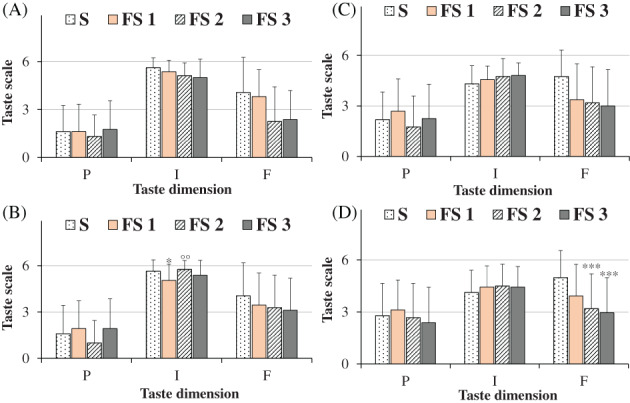
Ratings of pleasantness (P), intensity (I), and familiarity (F) dimensions determined for the taste of saline solutions obtained with salt (S, 100% sodium chloride) and flavored sea salts of myrtle (FS 1), Mediterranean herbs/plants (FS 2), and orange/saffron (FS 3) at the concentrations of 0.1 g mL^−1^ in men (*n* = 26) (A) and women (*n* = 32) (B) and 0.04 g mL^−1^ in men (*n* = 26) (C) and women (*n* = 32) (D). Data are presented as mean values and standard deviations. ***P < 0.001, *P < 0.05 *versus* S, °*P* < 0.01 *versus* FS 1 (one‐way ANOVA and the Bonferroni *post hoc* test).

The ratings of pleasantness, intensity, and familiarity dimensions determined for the taste of saline solutions (0.1 and 0.04 g mL^−1^) obtained with the three types of flavored sea salt (FS 1, FS 2 and FS 3), with respect to pure salt (100% sodium chloride), were reported for all participants in Fig. 5 and for men and women in Fig. 6. In general, at the high dose (Fig. 5(A)) salt and flavored sea salts were quite similar in terms of taste pleasantness and intensity dimensions, the latter characterized by a very high score (near to 6). Whereas all flavored sea salts showed lower scores in familiarity dimension than salt (Fig. 5(A)). At this dose, taste pleasantness of all salts (S, FS 1, FS2 and FS 3) was negatively correlated to taste intensity, as indicated by the correlation coefficient taste pleasantness/intensity (*r* = −0.7308). At the low dose (Fig. 5(B)), salt and flavored sea salts were similar in terms of taste pleasantness, while all flavored salts showed significant lower scores in familiarity dimension than salt (*P* < 0.01 for FS 1; *P* < 0.001 for FS 2 and FS 3, *versus* salt) (Fig. 5(B)). Moreover, evident differences, unless not significant, were determined in taste intensity scores, that followed the order: S < FS 1 < FS 2 ≅ FS 3, indicating the highest perception of salty taste intensity in solutions obtained with the sea salts flavored with a mixture of herbs/plants and oranges/saffron, characterized by 15% and 30% of flavoring extract, respectively. Interestingly, a negative correlation (*r* = −0.7303) was found between intensity of tested salts *versus* the extract percent amount (from 0% to 30%). A negative correlation was also found between intensity/familiarity of tested salts (*r* = −0.9703), indicating that the taste of saline solution obtained with pure salt was less intense but more familiar. Similar ratings of taste pleasantness, intensity and familiarity dimensions were determined at both doses in men and women for the three flavored sea salts (FS 1, FS 2, and FS 3) (Fig. 6), indicating in our experimental conditions no evident sex differences in the perception of salt taste dimensions.

Participants were also asked to provide a free description of the subjective sensory properties (flavor) of saline solutions (at the doses of 0.1 and 0.04 g mL^−1^) obtained with salt (sodium chloride; S) and the three flavored sea salts (FS 1, FS 2, and FS 3) and results are listed in Table 4. Saline solutions obtained from flavored salts were well distinguishable from those obtained from pure sodium chloride in terms of the presence of a specific flavor (indicated as note) or aftertaste (sensation still present in the mouth 1 min after swallowing).^43^ In general participants (untrained panelists) showed difficulties to individuate the exact flavoring agent (herb/spice) or identify the different components of the flavoring mixture. The sensory properties/flavor of product FS 1 and FS 2 were perceived as very similar, and the presence of rosemary extract was individuated in FS 2. The occurrence of myrtle extract was not recognized in FS 1, while for the product FS 3, the presence of orange and saffron extracts was individuated.

**Table 4:** The sensory evaluation of the taste of saline solutions, at the concentrations of 0.1 g mL^−1^ and 0.04 g mL‐1, obtained with normal sea salt (S) and different type of flavored sea salt: salt of myrtle (FS 1), Mediterranean herbs/plants (FS 2), and orange/saffron (FS 3)

	Sensory perceived attributes	
Salt type	High dose (0.1 g mL^−1^)	Low dose (0.04 g mL^−1^)
S	Sea water, strong salty taste with a note of sour taste, metallic taste	Sea water
FS 1	Strong salty taste, rosemary note, spicy note, rosemary aftertaste, origan aftertaste, spicy aftertaste, sweet herb aftertaste, bitter aftertaste, mint aftertaste	Rosemary note, spicy note, thyme note, caper note, rosemary aftertaste, origan aftertaste, spicy aftertaste
FS 2	Rosemary note, aromatic herbs note, pepper note, origan note, rosemary aftertaste, cumin aftertaste, origan aftertaste, turmeric aftertaste, spicy aftertaste, pepper aftertaste	Fennel note, rosemary note, thyme note, spicy aftertaste, rosemary aftertaste, curry aftertaste, origan aftertaste, meat aftertaste
FS 3	Strong salty taste, orange note, turmeric note, rosemary note, paprika note, orange aftertaste, saffron aftertaste, turmeric aftertaste, citrus fruits aftertaste, spicy aftertaste, lemon aftertaste	Rosemary note, turmeric note, orange aftertaste, citrus fruits aftertaste, rosemary aftertaste, aromatic herbs aftertaste, spicy aftertaste

Salty taste is the basic taste and saltiness is a specific sensation associated with a sodium chloride solution (in particular Na^+^).^43^ Low‐medium concentrations of table salt (sodium chloride), the common sodium‐containing chemical used to season foods, are perceived as pleasant and appetitive.^43^ We evaluated the sensory profiles of flavored sea salts in order to determine if the flavoring of salt with aromatic plants could impact the perceived saltiness. We observed that the flavoring of sea salt with Mediterranean herbs, spices, and fruits generally increased the perception of salty intensity. The dose of 0.1 g mL^−1^ emerged as too high to appreciate the sensory differences, in terms of taste intensity, among various saline solutions, with score values near to 6 for all type of salt. However, at the dose of 0.04 g mL^−1^, saline solutions obtained with flavored salts (containing approximately 6–30% less sodium chloride) were perceived as more intense but equally pleasant than pure salt solution. In particular, the oranges/saffron (FS 3) salt emerged as the most interesting in potentiating saltiness perception (considering the 30% reduction of salt weight). The lower scores in taste familiarity (indicating a less common taste) measured with saline solutions obtained with flavored salts also confirmed the modulation of salty perception induced by flavoring. Flavoring using blends of herbs and spices has been proved as strategy to reduce the salt content in food products.^5^, ^16^, ^17^, ^23^, ^44^ Herbs and spices have been demonstrated to enhance liking of low‐salt tomato soup,^17^ low‐salt legume‐based dishes,^17^ and chicken pasta meal.^23^ Together with amino acids, hydrolysates of proteins, nucleotides, and seaweed, spices/herbs are used as salt enhancers in food products.^44^ The multisensory interaction between aroma and taste can be employed to compensate for the lower salt levels in food products.^5^, ^44^

In the last years, a remarkable increase has been observed in the culinary use of herbs/spices‐flavored salts for their ability to provide complex flavor to foods. We presented evidence that addition of flavored salts instead of normal salt during food preparation/cooking could greatly contribute to a reduction of salt intake in daily diet without loss of saltiness perception and maybe food palatability. Flavor involves the combination of gustative perception of soluble and non‐volatile compounds (basic tastes), volatile compounds perceived through retronasal olfaction (aroma), and chemical sensations through the trigeminal nerve.^45^ Therefore, volatile compounds measured in *n*‐hexane extracts and polar components^26^ previously identified could therefore contribute/interfere to the taste/flavor of flavored sea salt solutions and saltiness perception. Polar compounds (phenols, sugars, and amino acids) of plant extracts incorporated in flavored sea salt could dissolve in water and maybe modulate the taste perception on sensory end‐organs (taste buds)^43^ present on the tongue. Whereas aromatic compounds present in flavored sea salt, liberated in the mouth, could be responsible of the flavor attributes^46^ indicated by the untrained panelists through retronasal olfaction.^45^

## CONCLUSIONS

Our study confirmed the important role of Mediterranean aromatic plants in the enhancement of saltiness perception and qualified the use of flavored sea salts during food preparation/cooking in place of normal salt as a potential strategy to reduce the daily salt intake. Further studies are needed in order to explore the best ratio/type of spices/herbs blends to be used in salt flavoring for domestic and industrial applications.

## CONFLICT OF INTEREST STATEMENT

The authors declare no conflict of interest.
